# Hepatitis C testing in substance use disorder treatment: the role of program managers in adoption of testing services

**DOI:** 10.1186/s13011-016-0057-2

**Published:** 2016-04-01

**Authors:** Jemima A. Frimpong, Thomas D’Aunno

**Affiliations:** Department of Health Policy and Management, Mailman School of Public Health, Columbia University, 722 West 168th Street, New York, NY 10032 USA; Robert F. Wagner Graduate School of Public Service, New York University, 295 Lafayette St., #3062, New York, NY 10012 USA

**Keywords:** Hepatitis C (HCV), Opioid treatment programs, Best practices, Adoption, Program managers, Management, Substance use disorder treatment

## Abstract

**Background:**

Health care organizations do not adopt best practices as often or quickly as they merit. This gap in the integration of best practices into routine practice remains a significant public health concern. The role of program managers in the adoption of best practices has seldom been investigated.

**Methods:**

We investigated the association between characteristics of program managers and the adoption of hepatitis C virus (HCV) testing services in opioid treatment programs (OTPs). Data came from the 2005 (*n* = 187) and 2011 (*n* = 196) National Drug Abuse Treatment System Survey (NDATSS). We used multivariate regression models to examine correlates of the adoption of HCV testing. We included covariates describing program manager characteristics, such as their race/ethnicity, education, and their sources of information about developments in the field of substance use disorder treatment. We also controlled for characteristics of OTPs and the client populations they serve.

**Results:**

Program managers were predominantly white and female. A large proportion of program managers had post-graduate education. Program managers expressed strong support for preventive services, but they reported making limited use of available sources of information about developments in the field of substance use disorder (SUD) treatment. The provision of any HCV testing (either on-site or off-site) in OTPs was positively associated with the extent to which a program manager was supportive of preventive services. Among OTPs offering any HCV testing to their clients, on-site HCV testing was more common among programs with an African American manager. It was also more common when program managers relied on a variety of information sources about developments in SUD treatment.

**Conclusions:**

Various characteristics of program managers are associated with the adoption of HCV testing in OTPs. Promoting diversity among program managers, and increasing managers’ access to information about developments in SUD treatment, may help foster the adoption of best practices.

## Background

Approximately 3.2 million people in the United States are living with chronic Hepatitis C virus (HCV) [1]. HCV is the most common blood-borne infection in the United States and recently surpassed HIV as a cause of death in the nation [2]. An estimated 50–75 % of chronically infected people remain unaware of their HCV infection [3]. The prevalence of chronic HCV infection among at-risk populations, especially, persons who inject drugs (PWID), ranges from 35–65 % [1, 4]. Availability of HCV testing is imperative to increasing awareness of infection status and setting the HCV continuum of care in motion [5]. HCV testing is recommended for all persons who have ever used illicit injection drugs or shared equipment used to prepare or inject drugs [6–8]. Even though addressing the HCV epidemic does not constitute a core mission of substance use disorder (SUD) treatment programs, systematically offering HCV testing in SUD treatment programs could help greatly reduce the proportion of HCV-infected individuals who are unaware of their infection [9, 10].

Yet, the integration of HCV testing into the practices of SUD treatment programs is incomplete [11, 12]. Even though the proportion of SUD treatment programs that offer HCV testing options to their clients has increased in recent years, missed opportunities for HCV testing remain [13, 14]. SUD treatment programs increasingly refer their clients to off-site facilities for HCV testing – a practice associated with significant reductions in the use of recommended services [14–16].

This increased use of off-site referrals for HCV testing is linked to constraints and barriers SUD treatment programs face in offering HCV-related and other medical services to SUD treatment patients.

Several studies have thus emphasized the role of human and financial resources, as well as other organizational characteristics, in the adoption of HCV testing. For example, the increasing use of off-site referrals for HCV testing was associated with declining federal funding in SUD treatment programs, with the offer of HCV testing being lower in SUD treatment programs that primarily prescribe buprenorphine only (rather than methadone) for SUD treatment [14]. Provider characteristics, including training, knowledge and workload may also limit the offer of HCV testing in SUD treatment programs [17, 18]. Some SUD treatment programs may not have phlebotomists on staff, and thus would need to hire new personnel before implementing HCV testing on-site. Performing HCV testing may exert further pressure on staff time, and significantly add to an already heavy workload. Other challenges to offering medical services into SUD treatment programs have also been highlighted [10, 19–21]. Implementing HCV testing may require authorizations and accreditations that some SUD treatment programs may not have. And finally, offering HCV testing may be perceived as peripheral to the primary mission of SUD treatment programs (i.e., the treatment of addiction and substance use disorders). Such factors may thus limit the integration of HCV-related services into SUD treatment services [21, 22].

Leadership and management of a SUD treatment program may also constitute an essential element of integrating HCV prevention and care services into SUD treatment [23]. Various studies of organizations in other areas of healthcare indicate that managers play an important role in defining the practices of an organization [24–26]. One of the more important decisions that managers make is to adopt relatively new evidence-based services or practices, seeking to improve their organization’s quality of care and outcomes for clients. This decision is especially critical in smaller health care organizations such as SUD treatment programs, in which managerial decisions have more direct and immediate effects than in larger organizations [27–30]. However, the role played by managers of SUD treatment programs (henceforth, managers) in offering HCV testing has seldom been investigated [9, 25, 31], even though managers can be an integral link between policy and practice, and can play a central role in the decision to adopt recommended practices [27, 32].

We hypothesize that three key characteristics of managers are related to their organizations’ adoption of HCV testing: (1) the demographic characteristics of managers (i.e., gender, race/ethnicity, age, educational level); (2) the sources of information they use for learning about advancements in the field of SUD treatment; and (3) their support for preventive services. In other healthcare contexts, race/ethnicity, [33, 34] age, [35] gender [36–38] and educational levels [39–41] of managers have all been associated with organizational strategy, including the provision of prevention and outreach services. The managers’ sources of information have also been shown to influence the practices of an organization [26, 40, 42, 43]. Thus, this study aims to test whether these attributes of program managers in a sample of SUD treatment programs are also associated with the offer of HCV testing services. We focus on the nation’s opioid treatment programs (OTPs) because opioid use is strongly associated with injection drug use, the most common mode of HCV infection and transmission [44, 45].

## Methods

### Sampling frame and sample

This study draws from two waves of the National Drug Abuse Treatment System Survey (NDATSS). NDATSS is a nationally-representative survey, which examines the organizational structures and operating characteristics of the nation’s outpatient SUD treatment programs [46]. The analysis sample focused on all OTPs surveyed in 2005 and 2011. We defined an OTP as a physical facility with resources dedicated specifically to treating opiate dependence through methadone or buprenorphine (excluding primary care or physicians offices). Opioid treatment programs are a particularly important setting for HCV testing because opioid use (e.g., heroin) is associated with injection drug use, one of the main modes of HCV transmission. Thus, integrating HCV testing with substance use, especially in opioid treatment programs is of considerable importance [19, 20].

Because SAMHSA licenses all OTPs, it has a list that precisely identifies the entire US population of approved OTPs. In 2007, SAMHSA reported that there were 1,108 licensed OTPs in the US. By 2011, this number increased to 1,459, with about 304,000 opioid-dependent individuals receiving services each day. OTPs, in 2011, accounted for about twenty-six percent of all individuals enrolled in SUD treatment programs across the nation [47]. The 2005 round of the NDATSS included data from 187 OTPs, with an 88 % response rate [46]. For the 2011 round of the NDATSS, we contacted OTPs that participated in 2005. To ensure that the 2011 sample was nationally representative and had adequate statistical power however, we also contacted additional randomly selected OTPs from SAMHSA’s 2011 list. In total, 200 OTPs completed surveys in 2011, for an overall response rate of 86.6 % [48].

We assessed possible non-response bias resulting from twenty-two OTPs that refused to participate in the 2011 wave, as well as ninety OTPs with which initial contact was made, but due to time and budget constraints, follow-ups were not completed. We compared participating OTPs to the two types of non-participating OTPs along twenty key variables (e.g., ownership; offer of HIV or HCV testing) and did not find any statistically significant differences. We also found that there were no differences between OTPs that were interviewed both in 2005 and 2011 (*n* = 59), and the OTPs that we added to the sample in 2011 (*n* = 141). These results suggest that changes in HCV testing and characteristics of OTP managers between 2005 and 2011 are not due to the inclusion of new OTPs to the sample. In 2011, 4 participating OTPs did not provide information on the availability of HCV testing for their patients. In this paper, our analysis thus included 187 and 196 OTPs interviewed in 2005 and 2011, respectively, for a total of 383 observations.

### Data collection

The program manager and clinical supervisor of each OTP were asked to complete a telephone survey on treatment practices and program characteristics. The manager survey collected information on the demographic characteristics of managers and their sources of information, as well as information concerning organizational structure, ownership, finances, and accreditation of the OTP. Clinical supervisors provided information about patient characteristics, staff composition, and offer of ancillary services, including HIV and HCV testing. The Columbia University Institutional Review Board reviewed and approved the study. The Columbia University IRB protocol number for the study is IRB-AAAF3894.

### Data reliability and validity

We followed established methods that maximize reliability and validity in phone surveys [49]. These methods included: pre-testing the survey with a random sample of ten programs; providing training about our study for trained telephone interviewers; sending each program manager a cover letter explaining the study, along with web-based work-sheets that inform participants of the requested data that enables them to consult financial and administrative records prior to the call; and making a brief phone call to follow-up on the letter.

Further, as data were collected, we performed extensive computer reliability checks to signal interviewers of inconsistent or infeasible responses (e.g., % of patients with various demographic characteristics should sum to 100 %). Interviewers then worked with respondents to resolve inconsistencies. Results were further scrutinized for reliability and validity. Reliability checks included comparisons of reported totals (e.g., total revenue) with the sum of reported detail (e.g., revenues by source); comparison of responses to related questions; comparison of responses between manager and supervisor; and, for panel programs, comparison of responses over time. The reliability and validity of the NDATSS, as well as detailed description of the sampling frame and sample are available from other sources [14, 48–50].

### Measures

#### Dependent variable

In each survey wave, managers were asked whether their staff routinely provided HCV testing services to their patients, and whether it was provided on-site or through off-site referrals. We constructed two dependent variables drawn from these questions. First, we examined whether programs had adopted any HCV testing option (i.e., either on-site or off-site offer of HCV testing). We created a variable set to 1 if the OTP offered HCV testing services either on-site or off-site (any HCV testing) and 0 otherwise (i.e., no HCV testing option). Second, we examined the adoption of on-site HCV testing among programs that offer any HCV testing. Offering HCV testing services on-site in OTPs could help increase case finding and reduce transmission [51, 52]. A recent study showed, for example, that on-site offer of HIV testing was associated with significant increase in the uptake of HIV testing [16]. Considering similarities in risk factors, modes of transmission and approaches to testing for HIV and HCV, we also examine the offer of HCV testing on-site in treatment programs, and the extent to which managerial characteristics are associated with on-site testing. We thus created another variable set to 1 if an OTP provided on-site HCV testing and 0 otherwise.

#### Predictor variables

Our main predictors of interest concern OTP managers. Race/ethnicity of managers was categorized as White, African-American, or Other. The “other” race/ethnicity category included American Indian or Alaskan Native, and Asian American or Pacific Islander. We categorized managers’ education as post-graduate training/professional schooling after college or college graduate or less. Managers’ access to information about developments in SUD treatment practices was measured by the extent to which they: (1) read journals and professional publications; (2) participated in seminars and workshops; (3) attended conferences; (4) held professional association memberships; (5) participated in advisory boards and commissions; and (6) engaged in informal conversations with colleagues outside their OTP. Responses to each question ranged from “no extent” (1) to “a very great extent” (5). Responses for the six questions were summed and divided by the number of questions answered, thus creating an “information index” with scores ranging from 1 (program manager does not use any external sources of information) to 5 (program manager uses all the available sources of information). Managers reported their support for several preventive services, including: (1) distribution of clean needles for their client; (2) distribution of bleach solutions to intravenous drug users to clean needles; (3) distribution of condoms to encourage safe sexual practices; and (4) support for needle exchange programs. Response categories ranged from “no extent” (1) to “a very great extent” (5). Managers’ responses to the four questions were summed and then divided by the number of questions, thus generating a support for preventive services construct with scores ranging from 1 (manager does not support the use of any preventive services) to 5 (manager supports the use of all available preventive services). An average alpha coefficient for source of information (α = 0.70) and support for preventive services (α = 0.86) indicate acceptable reliability of the constructs [53].

### Patient characteristics

We also included patient characteristics as control covariates in our analyses, since prior analyses suggest may these variables may confound the observed association between managers’ characteristics and the adoption of HCV testing [13, 14]. Patient characteristics included information on the mix of patient characteristics in each OTP. The clinical supervisor of each treatment program reported aggregated socio-demographic characteristics of patients, including the proportion of patients from the most recent complete fiscal year who were Non-Hispanic African-American or Hispanic/Latino; the proportion of female patients; and the proportion of patients who inject drugs. Other patient-related variables included percent of patients requiring prior authorization from their insurance provider before service provision.

### Program characteristics

OTP characteristics included a series of variables, which we have previously investigated in relation to HCV testing practices in OTPs [14]. We thus controlled for OTP ownership (public/private-for-profit/private-not-for-profit), as publicly owned OTPs might be more likely to see HCV testing as falling within their core mission of promoting public health. We included a variable indicating whether the Commission on Accreditation of Rehabilitation Facilities (CARF) accredited a treatment program. CARF accreditation is an indicator of quality of services provided by health service organizations. The use of buprenorphine for opioid abuse treatment has increased in recent years, and we assessed if OTPs’ use of buprenorphine might be related to their provision of HCV testing services. We thus included indicators for whether an OTP provided methadone maintenance treatment only; buprenorphine only; or methadone and buprenorphine [48]. We also controlled for OTPs financial resources. We included variables indicating whether programs received revenues from private insurance (none vs. at least 1 %) or federal funding sources (none vs. at least 1 %). Finally, we controlled for human resources available for HCV testing by including a measure of the patient to staff ratio. We divided the number of patients served by the program during in the most recent complete fiscal year, by the number of full time equivalent staff working 35 h a week or more employed by the program during the same period.

### Data analysis

We first described the characteristics of program managers in 2005 and 2011. We tested for changes in those characteristics between the two time points using *χ*^2^ tests for categorical variables and t-tests for continuous variables. We then estimated the association between managers’ characteristics and HCV testing practices using multivariate logistic regression models. We began by testing for possible interactions between each of the independent variables and a dummy variable denoting survey year for both dependent variables (i.e.,. any HCV testing and on-site HCV testing). We found that the association between only 3 managers’ characteristics and the likelihood of any HCV testing in an OTP changed significantly between 2005 and 2011. On the other hand, there were no significant changes in association of managers’ characteristics and on-site HCV testing from 2005 – 2011. We thus decided to pool observations from 2005 and 2011 for multivariate analysis. In these models, we controlled for patient and program characteristics. We also included a variable denoting survey year to control for time trends in the provision of HCV testing services in OTPs.

In multivariate models, the first dependent variable was whether a treatment program offered any HCV testing service, i.e., either on-site or off-site. The second dependent variable examined predictors of on-site testing among programs that offered any HCV testing. Some independent variables (percent of clients requiring prior authorization, and percent clients who are female) displayed missing observations in particular survey waves. When a given OTP displayed missing values for these variables, we imputed values by calculating predicted values using multiple regression analysis based on variables for NDATSS survey wave and the observed values of these variables within the same OTP program in other waves. We compared results obtained with imputed and non-imputed data and found that imputation had no substantive impact on our point estimates but increased our sample size in pooled regression analysis from 353 to 383 programs. We used the Huber-White sandwich estimators of the standard errors to take into account non-independence of observations within programs. We report odds ratios (OR) and 95 % Confidence Intervals (CI) for each dependent variable. All analyses were performed with Stata Version 12 [54].

## Results

### Descriptive statistics

Table 1 describes changes in HCV testing practices and the characteristics of OTP managers between 2005 and 2011. The proportion of OTPs that had adopted any HCV testing increased significantly from 73 % in 2005 to 90 % in 2011. However, during the same period, there was a substantial and statistically significant decline in on-site HCV testing among programs offering any HCV testing, from 53 to 34 % [14].

**Table 1:** Characteristics of Opioid Treatment Programs (OTPs)

	Full Sample	2005	2011	
	(N=383)	(N=187)	(N=196)	p-value*
Dependent Variables				
Any HCV testing				0.000
HCV testing (on-site or off-site)	81.7	73.3	89.8	
No HCV testing	18.3	26.7	10.2	
On-site vs. off-site HCV testing^+^				0.000
On-site HCV testing	52.7	72.3	37.5	
Off-site HCV testing only	47.3	27.7	62.5	
Predictor variables				
Director race/ethnicity				
African-American	14.9	14.4	15.3	0.812
Other race/ethnicity	8.4	11.2	5.6	0.047
White	76.7	74.7	79.1	0.313
Director age (mean, SD)	49.7 (9.6)	48.4 (8.5)	51 (10.4)	0.009
Director gender				0.354
Female	55.3	52.9	57.6	
Male	44.7	47.1	42.4	
Director education				0.156
Post-graduate training or more	70.8	67.4	74.0	
College or less	29.2	32.6	26.0	
Director sources of information (mean, SD)	3.2 (0.5)	3.3 (0.5)	3.1 (0.5)	0.001
Director support of preventive services (mean, SD)	3.9 (0.9)	3.7 (1.0)	4.0 (0.9)	0.004
Control variables				
*Patient characteristics (mean, SD)*				
Percent African-American patients	22.3 (24.9)	25.5 (26.5)	19.3 (22.9)	0.015
Percent Hispanic / Latino patients	16.4 (21.2)	17.9 (22.7)	15.0 (19.7)	0.189
Percent female patients	41.3 (13.6)	39.9 (15.8)	42.7 (11.0)	0.039
Percent persons who inject drugs	42.4 (29.8)	44.8 (30.9)	40.2 (28.6)	0.133
*Program characteristics*				
CARF accreditation				0.165
Yes	49.1	45.5	52.5	
No	50.9	54.5	47.5	
Ownership				0.150
Public	14.1	17.6	10.7	
Private for-profit	37.9	36.4	39.3	
Private not-for-profit	48.0	46.0	50.0	
Method of treatment				0.000
Both (Meth + Bup)	57.2	68.0	47.0	
Buprenorphine only (Bup)	24.3	19.2	29.1	
Methadone only (Meth)	18.5	12.8	23.9	
Staff - patient ratio (mean, SD)	0.04 (0.03)	0.04 (0.04)	0.05 (0.03)	0.025
Prior authorization (mean, SD)	22.32 (33.8)	19.20 (31.9)	25.30 (35.3)	0.078
Revenue from federal sources				0.004
At least 1%	28.5	37.9	21.5	
None	71.5	62.1	78.5	
Revenue from private insurance				0.142
At least 1%	42.3	41.1	44.3	
None	57.7	58.8	55.6	

Continuous data are mean ± Standard Deviation (SD). Categorical data are percentage

^+^Among OTPs that offer any HCV testing; n = 313 OTPs

*Denotes significant differences (p<. 05) between 2005 and 2011

OTP program managers were predominantly white, both in 2005 and 2011. White managers accounted for 75 and 79 % of program managers in 2005 and 2011, respectively. African-American managers represented roughly 15 % of OTP managers, both in 2005 and 2011. The proportion of OTP program managers from other races declined between 2005 and 2011, from 11.2 to 5.6 %. The average age of an OTP manager increased from 48.4 years in 2005 to 51.0 years in 2011. Program managers were predominantly women, both in 2005 and 2011. In 2011, for example, 57.6 % of program managers were women. The majority of OTP program managers had post-graduate training (67.4 % in 2005 and 74.0 % in 2011).

OTP program managers reported making limited use of sources of information about SUD treatment. Their average information score even declined between 2005 and 2011, from 3.3 to 3.1 (*p* = 0.001). On the other hand, OTP program managers expressed strong support for preventive services. Their preventive support score increased from 3.7 in 2005 to 4.0 in 2011 (*p* = 0.004).

### Multivariate results

Tables 2 and 3 show results from the multivariate analyses of the predictors of HCV testing practices in OTPs. We focus here on the association between characteristics of program managers and HCV testing. The association between clients or organizational characteristics and HCV testing practices have been described in detail elsewhere [13, 14].

**Table 2:** Offer of any HCV testing services (offer of either on-site or off-site HCV testing) in Opioid Treatment Programs

	(Any HCV testing vs. No testing)
	OR (95% CI)
Predictor variables	
Director race/ethnicity	
African-American	1.98 (0.65, 5.96)
Other race/ethnicity	0.42 (0.13, 1.27)
White	1†
Director age	1.01 (0.97, 1.03)
Director gender	
Female	0.67 (0.37 1.18)
Male	1†
Director education	
Post-graduate training or more	0.57 (0.28, 1.16)
College or less	1†
Director sources of information	1.50 (0.87, 2.58)
Director support of preventive services	1.39 (1.02, 1.90)*
Control variables	
*Patient characteristics*	
Percent African-American patients	0.99 (0.97, 1.00)^
Percent Hispanic / Latino patients	1.03 (1.00, 1.04)*
Percent female patients	0.99 (0.96, 1.00)
Percent persons who inject drugs	1.00 (0.99, 1.01)
*Program characteristics*	
CARF accreditation	
Yes	1.53 (0.75, 3.10)
No	1†
Ownership	
Public	1.10 (0.43, 2.76)
Private for-profit	1.12 (0.53, 2.34)
Private not-for-profit	1†
Method of treatment	
Both (Meth + Bup)	6.41 (1.69, 24.1)*
Buprenorphine only (Bup)	1.32 (0.58, 2.99)
Methadone only (Meth)	1†
Staff - patient ratio	1.34 (0.91, 1.94)
Prior authorization	1.00 (0.98, 1.00)
Revenue from federal sources	
At least 1%	1.40 (0.71, 2.73)
None	1†
Revenue from private insurance	
At least 1%	0.67 (0.33, 1.33)
None	1†
Year	
2005	0.30 (0.15, 0.58)***
2011	1†

A total of 383 OTPs were included in the analysis

Ninety-five percent confidence intervals estimated with robust standard errors

† Reference category; OR, Odds ratio; CI, Confidence interval

^p< 0.10. *p < 0.05. **p < 0.01.

**Table 3:** Offer of on-site HCV testing services among Opioid Treatment Programs offering any HCV testing (either on-site or off-site)

	(On-site vs. Off-site testing)
	OR (95% CI)
Predictor variables	
Director race/ethnicity	1†
African-American	2.76 (1.03, 7.35)*
Other race/ethnicity	0.66 (0.19, 2.27)
White	1†
Director age	0.97 (0.94, 1.00)^
Director gender	
Female	1.17 (0.65, 2.07)
Male	1†
Director education	
Post-graduate training or more	1.04 (0.55, 1.95)
College or less	1†
Director sources of information	2.35 (1.39, 3.97)**
Director support of preventive services	1.06 (0.78, 1.45)
Control variables	
*Patient characteristics*	
Percent African-American patients	0.99 (0.97, 1.00)
Percent Hispanic / Latino patients	1.01 (0.99, 1.02)
Percent female patients	1.01 (0.99, 1.03)
Percent persons who inject drugs	0.99 (0.98, 1.00)
*Program characteristics*	
CARF accreditation	
Yes	0.63 (0.31, 1.24)
No	1†
Ownership	
Public	3.05 (1.20, 7.72)*
Private for-profit	1.32 (0.62, 2.80)
Private not-for-profit	1†
Method of treatment	
Both (Meth + Bup)	2.10 (0.98, 4.48)^
Buprenorphine only (Bup)	0.14 (0.05, 0.33)***
Methadone only (Meth)	1†
Staff - patient ratio	1.70 (1.14, 2.54)*
Prior authorization	1.00 (0.99, 1.01)
Revenue from federal sources	
At least 1%	1.97 (0.98, 3.90)*
None	1†
Revenue from private insurance	
At least 1%	1.35 (0.70, 2.60)
None	1†
Year	
2005	4.74 (2.45, 9.13)***
2011	1†

A total of 313 OTPs were included in the analysis

Ninety-five percent confidence intervals estimated with robust standard errors

† Reference category; OR, Odds ratio; CI, Confidence interval

^p< 0.10. *p < 0.05. **p < 0.01. ***p < 0.001

Table 2 focuses on the association between program manager characteristics and the likelihood of an OTP offering any HCV testing option (either on-site or off-site) to their clients. After controlling for program and client characteristics, the demographic characteristics of a program manager were not associated with the likelihood of any HCV testing at an OTP. Similarly, a manager’s use of information sources about SUD treatment was not associated with her/his program’s offer of any HCV testing services. However, a manager’s support for preventive services in SUD treatment programs was positively associated with the likelihood of offering any HCV testing services (aOR = 1.39, 95 % CI = 1.02 to 1.90).

Table 3 focuses on the determinants of on-site HCV testing availability among OTPs that offer HCV testing options to their clients. Among those OTPs, those with African-American managers were significantly more likely to offer on-site HCV testing to their clients (OR = 2.76, 95 % CI: 1.03 to 7.35). OTPs whose program managers were older, on the other hand, were less likely to offer on-site HCV testing to their clients (aOR = 0.97, 95 % CI = 0.94 to 1.00). Other demographic characteristics of program managers were not associated with offer of on-site HCV testing. Managers’ use of information sources about SUD treatment was significantly associated with an increased likelihood of OTPs offering HCV testing on-site to their clients (aOR = 2.35, 95 % CI = 1.39 to 3.97). The attitudes of managers towards preventive services were not associated with offer of on-site HCV testing.

## Discussion

In this study, we investigated the association between the characteristics of managers of opioid treatment programs and the adoption of HCV testing practices. We found that key characteristics of managers were associated with adoption of HCV testing in OTP. In particular, a manager’s support for preventive services was associated with an increased likelihood that his/her organization offered at least one HCV testing option to its clients (either on-site or off-site). Previous studies report that attitudes and beliefs of program managers about preventive services are associated with their organizations’ use of best practices, including adequate methadone dose levels, HIV testing, and adoption of new pharmacotherapies [27, 46, 50]. Managers whose attitudes and beliefs favor preventive services as an essential component of effective SUD treatment may thus be more motivated to invest in, and provide, HCV testing services [55]. Similarly, programs whose managers used a larger number of information sources about SUD treatment were also more likely to offer on-site HCV testing. Efforts that increase exposure to a wide-variety of information sources among program managers may thus facilitate the adoption of HCV testing services [56].

Among programs that offered HCV testing to their clients, programs with an African-American manager were more likely to offer on-site HCV testing than other programs. A possible explanation for this association may be that African-American managers are more likely to work in communities with a higher proportion of minority clients and increased HCV prevalence [6]. These managers may therefore be more attuned to the provision of relevant services for reducing the burden of these diseases. This finding suggest that strategic recruitment of program managers with specific demographic profiles (e.g., racial/ethnic minorities) may help improve the adoption of testing practices, which could in turn have beneficial effects for all population groups. The Affordable Care Act (ACA) (Section 5307) provision of resources to support diversifying the healthcare workforce presents a promising mechanism for improving diversification of the health workforce [57]. Our work suggests that workforce diversity initiatives should also include strategies for diversifying the racial and ethnic composition of management teams [58].

Some of the characteristics of program managers we investigated may explain (part of) the increasing use of off-site referral for HCV testing in SUD treatment programs. This is the case of a manager’s age and her/his use of information sources about SUD treatment. The average age of OTP managers has increased significantly between 2005 and 2011. Since OTPs with older managers are less likely to offer on-site HCV testing, this trend may explain increasing use of off-site referrals for HCV testing. In addition, whereas the use of different information sources about SUD treatment is associated with on-site HCV testing, program managers reported using fewer sources in 2011 than they did in 2005. This decline in engagement with new developments in the field of SUD treatment may also have contributed to an increased reliance on off-site referrals for HCV testing.

### Limitations

There are limitations of the study that should be addressed. First, we examined a limited set of manager characteristics: demographic characteristics, sources of information for finding out about developments in the SUD treatment field, and support of preventive services. There are other factors, such as clinical training, discipline, tenure in the field of SUD treatment, attitudes toward the adoption of innovations, and innovation compatibility, which may influence treatment practices [59, 60]. The potential role of these characteristics should be investigated in future studies. Second, decision-making processes that influence the adoption of treatment practices are likely complex and may entail factors that were not included in the current analysis. In particular, aspects of teamwork and organizational climate were not included in these analyses. They may however mediate the effects of managers’ characteristics on the adoption of best practices such as HCV testing. Third, our estimates only measured the association between characteristics of program managers and HCV testing practices, and cannot be considered as causal. This is the case because program managers are purposefully recruited by OTPs, rather than randomly assigned to them. As a result, a number of managers’ characteristics may be systematically related to unobserved OTP characteristics that also affect the decision to offer HCV testing. Causal estimates of the impact of program managers’ characteristics on HCV testing would require either conducted a randomized trial of different managerial recruitment strategies. Improved estimates could also be obtained using panel data on the HCV testing practices of OTPs, which would facilitate examination of adoption and discontinuation of HCV testing. Unfortunately, the subset of OTPs that were interviewed both in 2005 and 2011 in NDATSS was too small to permit the latter analyses.

## Conclusions

Although the general management literature has emphasized the importance of managers and management practices on the adoption of best practices [35, 36, 61–64], the health services literature has not extensively examined these relationships [25, 27]. As such, there is a paucity of evidence linking manager attributes and treatment practices, especially in substance use disorder treatment programs. We find that the characteristics of program managers are an essential aspect of understanding the scope of services available to patients in opioid treatment programs. The current analysis presents preliminary empirical evidence that managerial characteristics are associated with the adoption of HCV testing, and could potentially influence patient outcomes. Our findings have important implications for strategies to promote adoption of HCV testing and other best practices in opioid treatment programs.

## References

[1] CDC. http://www.cdc.gov/hepatitis/Resources/Professionals/PDFs/ABCTable.pdf. Accessed 20 October 2012.

[2] Ly KN, Xing J, Klevens RM, Jiles RB, Ward JW, Holmberg SD (2012). The Increasing Burden of Mortality from Viral Hepatitis in the United States Between 1999 and 2007. Ann Intern Med.

[3] Mitchell AE, Colvin HM, Palmer Beasley R (2010). Institute of Medicine recommendations for the prevention and control of hepatitis B and C. Hepatology.

[4] Amon JJ, Garfein RS, Ahdieh-Grant L, Armstrong GL, Ouellet LJ, Latka MH, Vlahov D, Strathdee SA, Hudson SM, Kerndt P (2008). Prevalence of hepatitis C virus infection among injection drug users in the United States, 1994–2004. Clin Infect Dis.

[5] Viner K, Kuncio D, Newbern EC, Johnson CC (2015). The continuum of hepatitis C testing and care. Hepatology.

[6] Armstrong GL, Wasley A, Simard EP, McQuillan GM, Kuhnert WL, Alter MJ (2006). The prevalence of hepatitis C virus infection in the United States, 1999 through 2002. Ann Intern Med.

[7] Wasley A, Miller JT, Finelli L (2005). Surveillance for acute viral hepatitis--United States. Morb Mortal Wkly Rep Surveill Summ.

[8] Alter MJ, Seeff LB, Bacon BR, Thomas DL, Rigsby MO, Di Bisceglie AM (2004). Testing for hepatitis C virus infection should be routine for persons at increased risk for infection. Ann Intern Med.

[9] Glasner-Edwards S, Rawson R (2010). Evidence-based practices in addiction treatment: review and recommendations for public policy. Health Policy.

[10] Kresina TF, Hoffman K, Lubran R, Clark HW (2007). Integrating hepatitis services into substance abuse treatment programs: new initiatives from SAMHSA. Public Health Rep.

[11] Miller WR, Sorensen JL, Selzer JA, Brigham GS (2006). Disseminating evidence-based practices in substance abuse treatment: a review with suggestions. J Subst Abus Treat.

[12] Center for Substance Abuse Prevention (2009). Identifying and Selecting Evidence-Based Interventions Revised Guidance Document for the Strategic Prevention Framework State Incentive Grant Program. HHS Pub No (SMA)09-4205.

[13] Frimpong JA (2013). Missed opportunities for hepatitis C testing in opioid treatment programs. Am J Public Health.

[14] Frimpong JA, D’Aunno T, Jiang L (2014). Determinants of the Availability of Hepatitis C Testing Services in Opioid Treatment Programs: Results From a National Study. Am J Public Health.

[15] Fishbein DA, Lo Y, Reinus JF, Gourevitch MN, Klein RS (2004). Factors associated with successful referral for clinical care of drug users with chronic hepatitis C who have or are at risk for HIV infection. J Acquir Immune Defic Syndr.

[16] Metsch LR, Feaster DJ, Gooden L, Matheson T, Mandler RN, Haynes L, Tross S, Kyle T, Gallup D, Kosinski AS (2012). Implementing Rapid HIV Testing With or Without Risk-Reduction Counseling in Drug Treatment Centers: Results of a Randomized Trial. Am J Public Health.

[17] Mehta SH, Genberg BL, Astemborski J, Kavasery R, Kirk GD, Vlahov D, Strathdee SA, Thomas DL (2008). Limited uptake of hepatitis C treatment among injection drug users. J Community Health.

[18] Strauss SM, Astone-Twerell JM, Munoz-Plaza C, Des Jarlais DC, Gwadz M, Hagan H, Osborne A, Rosenblum A (2007). Correlates of drug treatment program staff’s self efficacy to support their clients’ hepatitis C virus (HCV) related needs. Am J Drug Alcohol Abuse.

[19] Litwin AH, Soloway I, Gourevitch MN (2005). Integrating Services for Injection Drug Users Infected with Hepatitis C Virus with Methadone Maintenance Treatment: Challenges and Opportunities. Clin Infect Dis.

[20] Kresina TF, Bruce RD, Lubran R, Clark HW (2008). Integration of viral hepatitis services into opioid treatment programs. J Opioid Manag.

[21] Hood KB, Robertson AA, Baird-Thomas C (2014). Implementing solutions to barriers to on-site HIV testing in substance abuse treatment: A tale of three facilities. Eval Program Plann.

[22] Haynes LF, Korte JE, Holmes BE, Gooden L, Matheson T, Feaster DJ, Leff JA, Wilson L, Metsch LR, Schackman BR (2011). HIV rapid testing in substance abuse treatment: Implementation following a clinical trial. Eval Program Plann.

[23] Birkhead GS, Klein SJ, Candelas AR, O’Connell DA, Rothman JR, Feldman IS, Tsui DS, Cotroneo RA, Flanigan CA (2007). Integrating multiple programme and policy approaches to hepatitis C prevention and care for injection drug users: A comprehensive approach. Int J Drug Policy.

[24] Silvia C, McGuire M (2010). Leading public sector networks: An empirical examination of integrative leadership behaviors. Leadersh Q.

[25] D’Aunno T (2006). The role of organization and management in substance abuse treatment: Review and roadmap. J Subst Abus Treat.

[26] D’Aunno T, Vaughn TE (1995). An organizational analysis of service patterns in outpatient drug abuse treatment units. J Subst Abuse.

[27] Friedmann PD, Jiang L, Alexander JA (2010). Top manager effects on buprenorphine adoption in outpatient substance abuse treatment programs. J Behav Health Serv Res.

[28] Young GJ, Charns MP, Shortell SM (2001). Top manager and network effects on the adoption of innovative management practices: A study of TQM in a public hospital system. Strategic Manage J.

[29] Guerrero EG (2010). Managerial capacity and adoption of culturally competent practices in outpatient substance abuse treatment organizations. J Subst Abus Treat.

[30] McConnell KJ, Hoffman KA, Quanbeck A, McCarty D (2009). Management practices in substance abuse treatment programs. J Subst Abus Treat.

[31] Garner BR (2009). Research on the diffusion of evidence-based treatments within substance abuse treatment: A systematic review. J Subst Abus Treat.

[32] McConnell KJ, Lindrooth RC, Wholey DR, Maddox TM, Bloom N (2013). Management practices and the quality of care in cardiac units. JAMA Intern Med.

[33] Putnam SM, Stiles WB, Jacob MC, James SA (1985). Patient exposition and physician explanation in initial medical interviews and outcomes of clinic visits. Med Care.

[34] Smedley BD, Stith AY, Nelson AR (2003). Unequal Treatment:Confronting Racial and Ethnic Disparities in Health Care (with CD).

[35] Kabacoff RI (2002). Leadership: What Has Age Got to do With It?.

[36] Jacobson WS, Palus CK, Bowling CJ (2010). A Woman’s Touch? Gendered Management and Performance in State Administration. J Public Adm Res Theory.

[37] Eagly AH, Johannesen-Schmidt MC (2001). The leadership styles of women and men. J Soc Issues.

[38] Burke S, Collins K (2001). Gender differences in leadership styles and management skills. Women Manage Rev.

[39] Wells R, Lemak CH, D’Aunno TA (2005). Organizational survival in the outpatient substance abuse treatment sector, 1988–2000. Med Care Res Rev.

[40] Wells R, Lemak CH, D’Aunno TA (2006). Insights from a national survey into why substance abuse treatment units add prevention and outreach services. Subst Abuse Treat Prev Policy.

[41] Chuang E, Wells R, Alexander R, Green S (2013). How outpatient substance abuse treatment unit director activities may affect provision of community outreach services. Drugs Educ Prevention Policy.

[42] Miller D, Vries MFRKD, Toulouse J-M (1982). Top Executive Locus of Control and Its Relationship to Strategy-Making, Structure, and Environment. Acad Manag J.

[43] Boeker W (1997). Strategic Change: The Influence of Managerial Characteristics and Organizational Growth. Acad Manag J.

[44] Des Jarlais DC, Semaan S (2008). HIV prevention for injecting drug users: the first 25 years and counting. Psychosom Med.

[45] Santibanez SS, Garfein RS, Swartzendruber A, Purcell DW, Paxton LA, Greenberg AE (2006). Update and overview of practical epidemiologic aspects of HIV/AIDS among injection drug users in the United States. J Urban Health.

[46] Pollack HA, D’Aunno T (2010). HIV testing and counseling in the nation’s outpatient substance abuse treatment system, 1995–2005. J Subst Abus Treat.

[47] SAMHSA. National Survey of Substance Abuse Treatment Services (N-SSATS): 2011. Data on Substance Abuse Treatment Facilities. BHSIS Series S-64, HHS Publication No. (SMA) 12-4730. Rockville, MD: Substance Abuse and Mental Health Services Administration; 2012.

[48] D’Aunno T, Pollack HA, Jiang L, Metsch LR, Friedmann PD (2014). HIV Testing in the Nation’s Opioid Treatment Programs, 2005–2011: The Role of State Regulations. Health Serv Res.

[49] Adams TK (2005). Outpatient Substance Abuse Treatment Surveys (OSATSS) sampling and weighting documentation for OSATSS-6, 2004. Survey Research Center, Institute for Social Research.

[50] Alexander JA, Wells R, Jiang L, Pollack H (2008). Organizational determinants of boundary spanning activity in outpatient substance abuse treatment programmes. Health Serv Manag Res.

[51] SAMHSA. Addressing Viral Hepatitis in People With Substance Use Disorders. In: Treatment Improvement Protocol (TIP) 53, SAMHSA CSAT; 2011. 22514861

[52] SAMHSA (2011). The TEDS Report: Injection Drug Abuse Admissions to Substance Abuse Treatment:1992 and 2009.

[53] Nunnaly J (1978). Psychometric theory.

[54] StataCorp (2012). Stata Statistical Software: Release 12.

[55] Astone JM, Strauss SM, Hagan H, Des Jarlais DC (2004). Outpatient drug treatment program directors’ hepatitis C-related beliefs and their relationship to the provision of HCV services. Am J Drug Alcohol Abuse.

[56] Gibbert WS, Keating SM, Jacobs JA, Dodson E, Baker E, Diem G, Giles W, Gillespie KN, Grabauskas V, Shatchkute A (2013). Training the workforce in evidence-based public health: an evaluation of impact among US and international practitioners. Prev Chronic Dis.

[57] DHHS. The Affordable Care Act, Section by Section. Title IV.http://www.hhs.gov/healthcare/rights/law/index.html. Accessed 10 February 2014.

[58] Guerrero EG (2013). Workforce diversity in outpatient substance abuse treatment: the role of leaders’ characteristics. J Subst Abus Treat.

[59] Blum TC, Davis CD, Roman PM (2014). Adopting evidence-based medically assisted treatments in substance abuse treatment organizations: roles of leadership socialization and funding streams. J Health Hum Serv Adm.

[60] Abraham AJ, Ducharme LJ, Roman PM (2009). Counselor attitudes toward pharmacotherapies for alcohol dependence. J Stud Alcohol Drugs.

[61] Carlile PR (2002). A pragmatic view of knowledge and boundaries: Boundary objects in new product development. Organ Sci.

[62] Hambrick DC (2007). Upper Echelons Theory: An Update. Acad Manag Rev.

[63] Hansen MT (1999). The Search-Transfer Problem: The Role of Weak Ties in Sharing Knowledge across Organization Subunits. Adm Sci Q.

[64] Tushman ML, Scanlan TJ (1981). Characteristics and external orientations of boundary spanning individuals. Acad Manag J.

